# Acyl Chain Length of Phosphatidylserine Is Correlated with Plant Lifespan

**DOI:** 10.1371/journal.pone.0103227

**Published:** 2014-07-24

**Authors:** Yan Li, Guowei Zheng, Yanxia Jia, Xiaomei Yu, Xudong Zhang, Buzhu Yu, Dandan Wang, Yanling Zheng, Xuejun Tian, Weiqi Li

**Affiliations:** 1 Key Laboratory of Biodiversity and Biogeography, Kunming Institute of Botany, Chinese Academy of Sciences, Kunming, Yunnan, China; 2 Plant Germplasm and Genomics Center, Germplasm Bank of Wild Species, Kunming Institute of Botany, Chinese Academy of Sciences, Kunming, Yunnan, China; 3 Department of Biology, Honghe University, Mengzi, Yunnan, China; University College Dublin, Ireland

## Abstract

Plant lifespan is affected by factors with genetic and environmental bases. The laws governing these two factors and how they affect plant lifespan are unclear. Here we show that the acyl chain length (ACL) of phosphatidylserine (PS) is correlated with plant lifespan. Among the detected eight head-group classes of membrane lipids with lipidomics based on triple quadrupole tandem mass spectrometry, the ACL of PS showed high diversity, in contrast to the ACLs of the other seven classes, which were highly conserved over all stages of development in all plant species and organs and under all conditions that we studied. Further investigation found that acyl chains of PS lengthened during development, senescence, and under environmental stresses and that increasing length was accelerated by promoted- senescence. The acyl chains of PS were limited to a certain carbon number and ceased to increase in length when plants were close to death. These findings suggest that the ACL of PS can count plant lifespan and could be a molecular scale ruler for measuring plant development and senescence.

## Introduction

Plant lifespan is a complex manifestation of genetic and environmental factors that encompass both development and senescence. It greatly influences crop productivity, biodiversity, and ecological equilibrium. Lifespan may be extended by enhancing resistance to internal or external stresses in yeast and animal, as described by the “stress resistance” theory of ageing [1], [2]. Telomere lengths can determine lifespan in mammals [3], [4], but this does not correlate with lifespan in plants [5], [6]. In *Arabidopsis*, for example, there was no significant difference in telomere length among the leaves at different stages of development [7]. Growth rings are widely used to measure the age of woody plants but they cannot predict the end of plant life. So far little is known about the determination of plant lifespan [8]. No change in primary structures of bio-molecules, DNA, proteins, saccharides, or lipids has been associated with development or lifespan of plants.

Glycerolipids are major constituents of cellular membranes. They comprise a glycerol backbone, two acyl groups, and a head group (Figure S1). The acyl groups contain two fatty acid chains at the *sn*-1 and *sn*-2 positions of the glycerol backbone. The two fatty acid chains of the glycerolipids in cellular membranes usually comprise fewer than 18 carbon atoms per chain or 36 carbon atoms in total. Fatty acids with more than 18 carbon atoms per chain are known as very long chains of fatty acids (VLCFAs). VLCFAs mainly distribute in cuticular waxes, aliphatic suberins, phospholipids, sphingolipids and seed oils [9]. PS is a head-group class of membrane glycerolipids which contains VLCFA [10], [11]. The glycerolipids in cellular membranes consist of multiple types of lipid molecule, which vary with respect to the head group, the lengths of the fatty acid chains, and the degree of unsaturation of the fatty acids. The cellular membranes in plants contain a highly diverse range of lipids, but the reasons for this diversity are not fully understood [12].

Using a lipidomics approach based on electrospray ionization and triple quadrupole tandem mass spectrometry (ESI-MS/MS) [11], [13], [14], we identified and quantified 140 molecular species of eight classes of membrane glycerolipid in nine plant species from five families. The plant species were chosen randomly. They comprised *Arabidopsis* and eight wild species that were distributed from alpine regions to maritime land areas and covered annual, biennial, and perennial species (Figure S2). Each molecular species was identified in relation to its total number of acyl carbon atoms and double bonds (Figure S3) [11], [13]. We calculated the molar percentage (mol%) of each molecular species on the basis of its content (nmol/mg) (Figure S3). Then, using the distribution of molecular species for each class, we calculated the average number of acyl carbon atoms for each class of head group (Figure S4).

## Materials and Methods

### Plant materials, growth conditions and treatments

We grew *Arabidopsis* ecotype Columbia (Col) and *Crucihimalaya* in soil, hydroponically using Hoagland's medium [15], and/or on plates in MS media, as indicated, at 22°C, under light of 120 µmol/m^2^/s, and a 12/12 h photoperiod. For phytohormone-induced senescence, we placed leaves that had been detached from 6-week-old plants onto filter paper that contained water, ABA (50 µM), or ethephon (50 µM, to release ethylene), and incubated them for the indicated number of days [16], [17]. To induce senescence via gamma radiation, hydroponically grown plants aged 20 days were irradiated in a commercial ^6^°Co facility (FJX-648G) at the indicated dose, grown under normal conditions, and sampled at the indicated times. To induce stress from heat shock, we incubated plates with plants aged 10 days in water at 44°C for 2 h [17]. To induce stress from dehydration, we placed plate-grown plants onto filter paper and exposed them to air for the indicated time [18]. All reagents described above were obtained from Sigma.

### Lipid analysis and data processing

We analysed lipid samples by ESI-MS/MS [13]. We processed the data in the same way that we have described in previous papers [14], [19]. We quantified the lipids in each class in comparison to two internal standards. The lipid content was described as nmol/mg dry weight of plants. We analysed five replicates of each plant species at each sampling time. Paired values were subjected to a *t* test to determine statistical significance. The average carbon number (C) of the acyl chains in a given lipid class was calculated using the formula: C =  (∑[*N* × mol% lipid])/100, where *N* is the total number of acyl carbons in each lipid molecule.

## Results

### ACLs of membrane glycerolipids in plants

**Figure 1:**
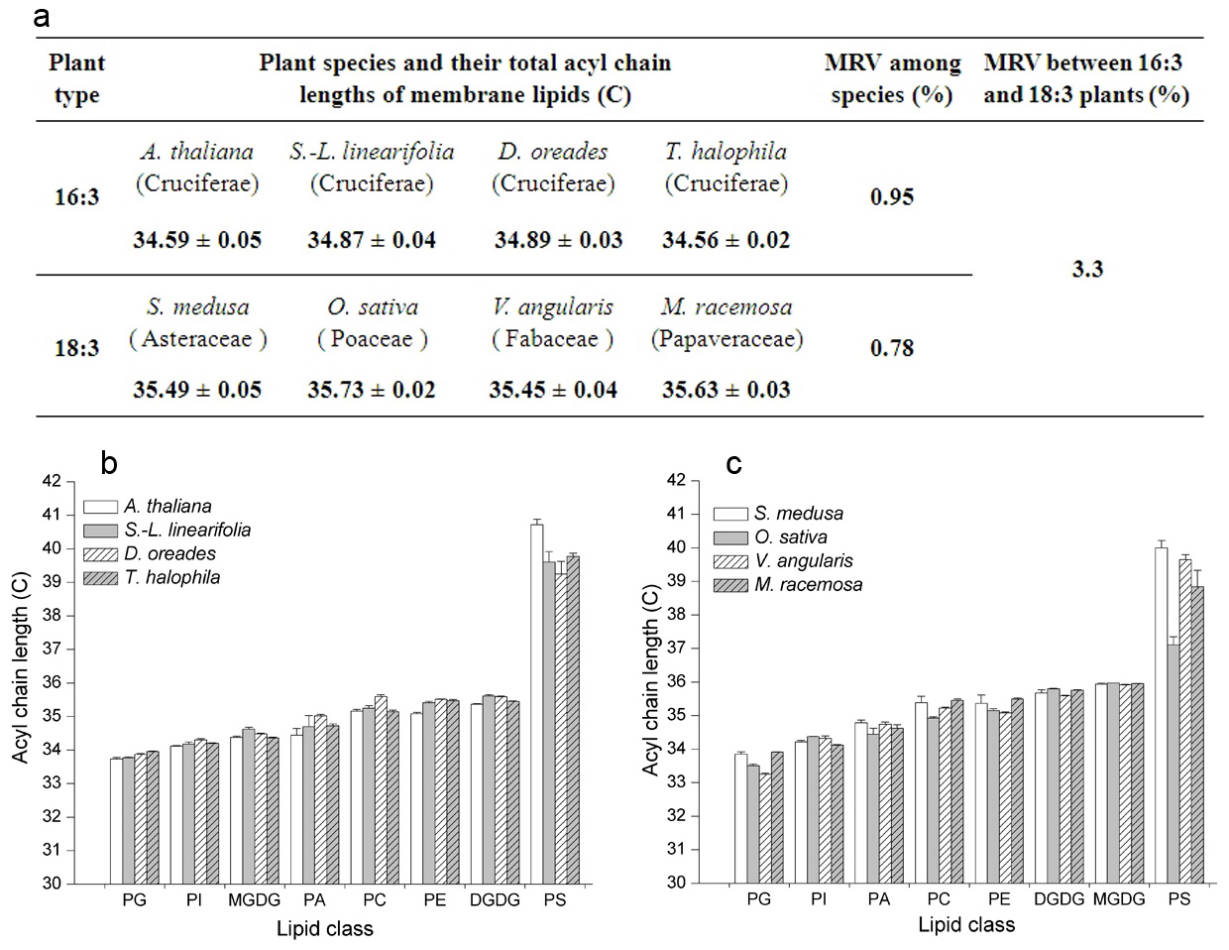
Acyl chain lengths of eight plants. (**a**) Acyl chain lengths of membrane lipids, (**b**) The acyl chain length of membrane glycerolipids of 16∶3 plants, or (**c**) The acyl chain length of membrane glycerolipids of 18∶3 plants. MRV, maximum relative variation. Values are means ± s.d. (*n* = 5).

We found that four species of Cruciferae contained 34∶6 monogalactosyldiacylglycerol (MGDG) molecular species, which indicated that they were 16∶3 plants (synthesizing lipids through both prokaryotic and eukaryotic pathways, Figure S5). In contrast, the four other species, which belonged to Asteraceae, Poaceae, Fabaceae, and Papaveraceae, did not harbour 34∶6 MGDG molecular species, which indicated that they were 18∶3 plants (synthesizing lipids through eukaryotic pathway, Figure S5). For the four 16∶3 plants, mean ACLs for all classes of glycerolipid were clustered in a very narrow range of 34.59–34.89 carbon (C); the maximum relative variation in ACL was 0.95%. For the four 18∶3 plants, the ACLs were clustered in the range 35.45–35.73 C, with a maximum relative variation of 0.78% (Figure 1a). The maximum relative variation of ACL among all eight species was only 3.3%; this variation resulted mainly from the different contribution of 34∶6 MGDG to the two types of plants (Figure S5). These results indicate that the ACL of membrane glycerolipids was highly conserved.

We then dissected the mean ACLs of membrane glycerolipids into each head-group class. We found that the ACLs of each class were distinctive, but their ranks based on length were the same in each plant type (Figure 1b and c). The variation in ACL was small for all lipid classes except PS (Figure S6). The variation in PS was 4.02 C, ranging from 36.70 to 40.72 C. The results indicated that the ACLs of MGDG, digalactosyldiacylglycerol (DGDG), phosphatidylglycerol (PG), phosphatidylethanolamine (PE), phosphatidylcholine (PC), phosphatidylinositol (PI), and phosphatidic acid (PA) were highly conserved but those of PS were highly diverse.

### Changes in ACL of PS during development

**Figure 2:**
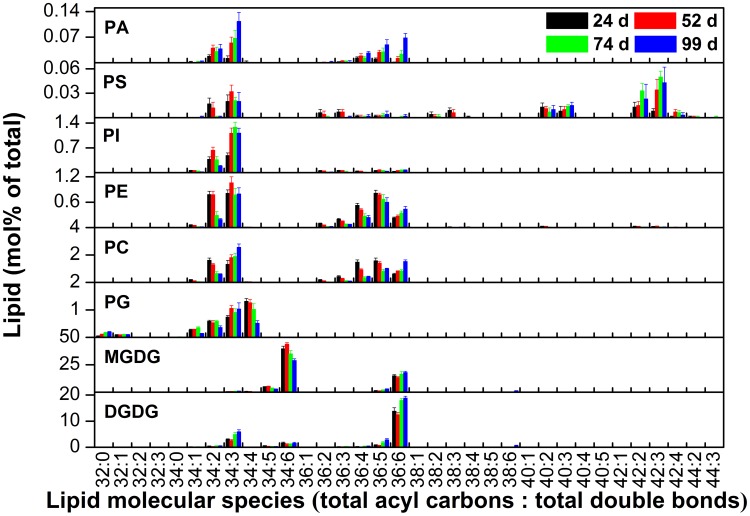
The relative composition (mol%) of lipid molecular species during development of leaves after germination. Values are means ±s.d. (*n* = 5).

**Figure 3:**
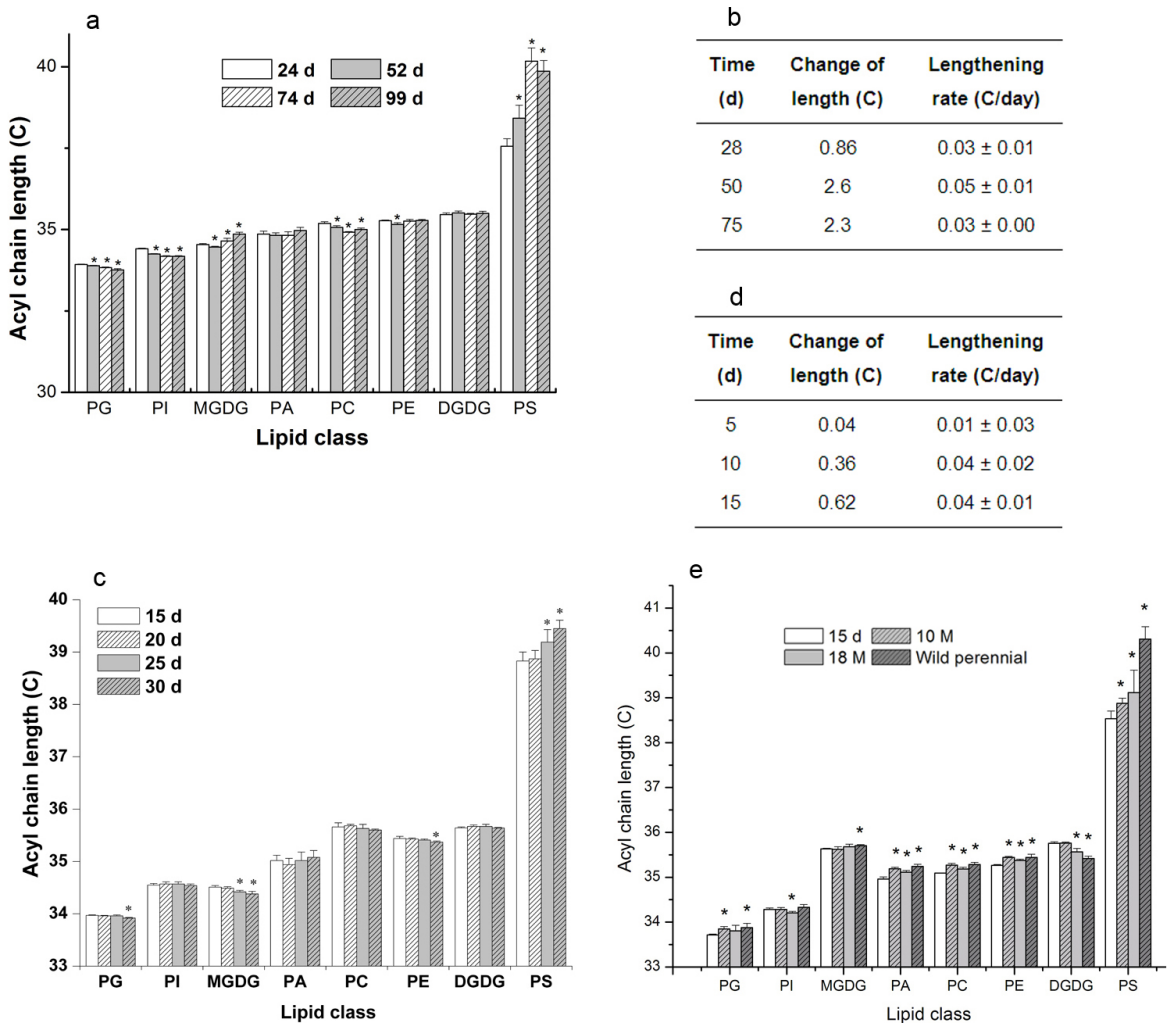
Acyl chains lengthen with plant development. (**a**) The acyl chain length of membrane glycerolipids during the development of leaves at 24, 52, 74 and 99 d in *Arabidopsis*, (**b**) The lengthening rate of acyl chains of PS during the development of leaves in *Arabidopsis*, (**c**) The acyl chain length of membrane glycerolipids during the development of siliques at 15, 20, 25 and 30 d in *Arabidopsis*, (**d**) The lengthening rate of acyl chains of PS during the development of siliques, (**e**) The acyl chain length of membrane glycerolipids during the development in roots of 15 day, 10 month, 18 month pot-grown, and wild perennial *C. himalaica* (W). Values are means ±s.d. (*n* = 5). An asterisk indicates that the value is different from that of the earliest stage of the development detected (*p<0.05*).

We sought to identify the factors that caused the diversity in ACL for PS and the biological significance of this diversity. Because the samples for the lipid analysis described above were collected from plants at different stages of development from various habitats, we hypothesized that ACLs of PS are affected by both developmental and environmental factors. To test the hypothesis, we first profiled lipid changes (Figure 2) and determined the ACLs of glycerolipids in *Arabidopsis* leaves during development from 24 to 99 days after germination, which covered the processes of growth, senescence, and death (Figure S7). The results showed that the ACLs for the seven lipid classes other than PS were highly conserved, and were clustered in a range of no more than 0.27 C. In contrast, the ACL for PS increased significantly by 2.6 C from 24 to 74 days, namely during the growth and senescence phases, and ceased to increase when the plants were dying (Figure 3a). The maximum rate of increase was 0.05±0.01 C/day up to about day 50 of the measured period of plant development (Figure 3b).

We then examined the ACL of PS in the main organs of plants to test whether an increase in the ACL of PS during development is a universal phenomenon in plants. We determined the ACLs for the eight classes of lipid in siliques at 15, 20, 25, and 30 days after flowering (Figure S8). The ACLs for DGDG, PC, PI, and PA remained unchanged over this period, and for MGDG, PG, and PE, decreased slightly by 0.1 C. In contrast, the ACL for PS increased significantly by 0.62 C (Figure 3c). The maximum rate of increase was 0.04±0.01 C/day during the period of silique development analysed (Figure 3d). We determined the ACL for PS in the roots of 15-day, 10-month, and 18-month pot-grown and wild perennial *Crucihimalaya himalaica* (Figure S2 and S9). The ACL for PS increased significantly by 1.78 C, whereas the ACLs for the other seven lipids remained within a narrow range (Figure 3e). These observations indicated that the acyl chains of lipids in the PS lengthened as development and that the maximum length was no more than 41 C.

### Changes in ACL of PS during senescence

**Figure 4:**
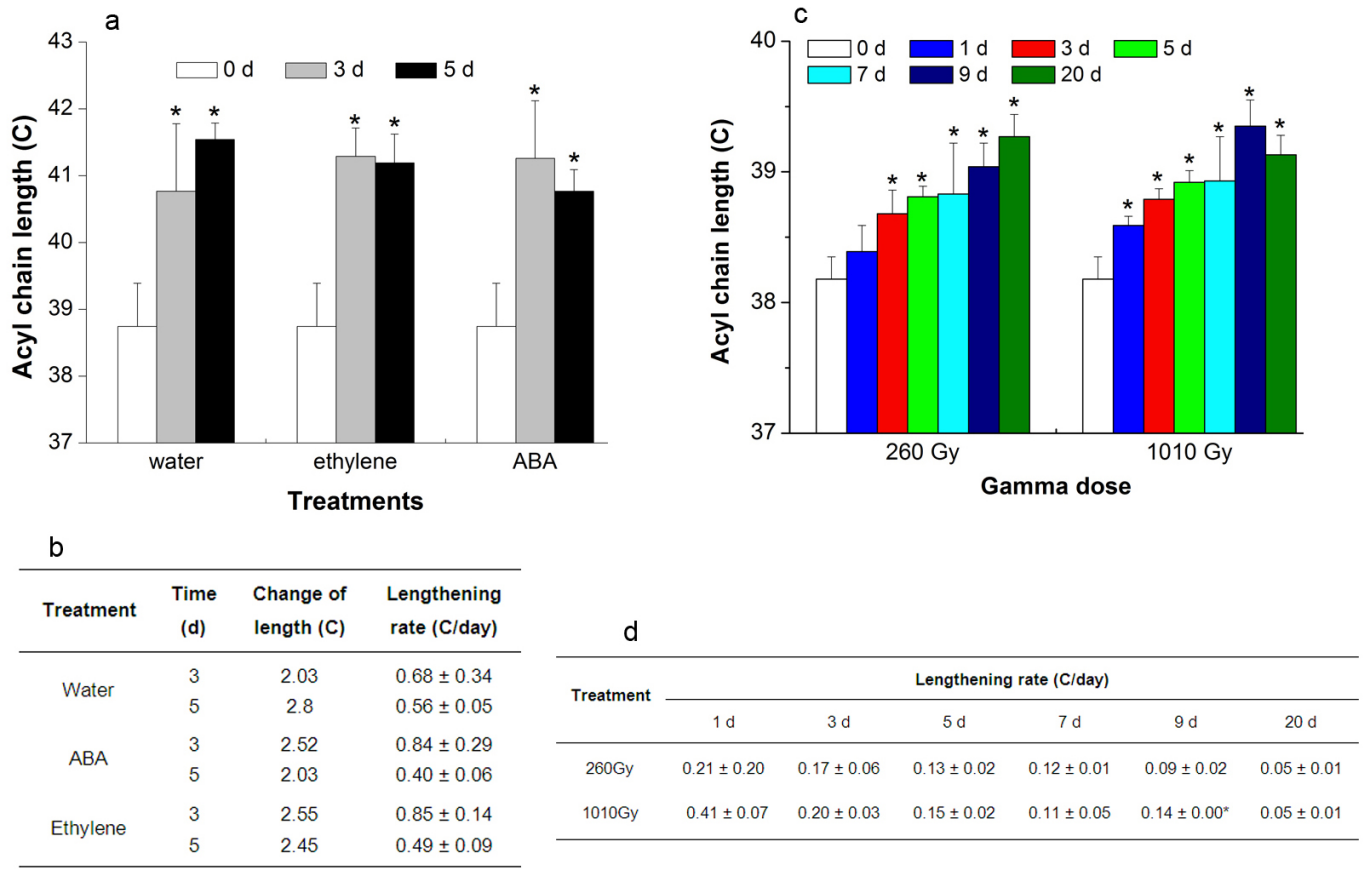
Acyl chains of PS lengthen with detachment-, ABA- and ethylene-induced senescence, and gamma irradiation in *Arabidopsis*. (**a**) The acyl chain length of PS treated with water, ABA, and ethylene in detached leaves, (**b**) The lengthening rate of PS treated with water, ABA, and ethylene in detached leaves, (**c**) The acyl chain length of PS in leaves at 0, 1, 3, 5, 7, 9, and 20 d after irradiation, (**d**) The lengthening rate of PS in leaves after irradiation. Values are means ±s.d. (*n* = 5). An asterisk indicates that the value is different from that of control or that of 260 Gy at same time (*p<0.05*).

Senescence can be induced in leaves by detaching them from the plant and the senescence of detached leaves can be accelerated by the application of the phytohormones abscisic acid (ABA) and ethylene[16]. We examined the changes in ACL of lipids during senescence at 0, 3, and 5 days after leaves were detached and treated with ABA or ethylene. Hormone-treated leaves showed yellowing on their edges in comparison with control, meaning their senescence was promoted (Figure S10). The results showed that the ACLs for PS significantly increased by more than 2 C in these three treatments (Figure. 4a), whereas in the other seven lipid classes, the ACLs were highly conserved (Figure S11). The maximum ACL for PS was ∼41.5 C. The maximum rate of lengthening was 0.68±0.34 C/day, which was significantly higher than that under normal growth conditions (Figures. 3b and 4b). Thus, it shows that the acyl chains of PS lengthened during detachment- and phytohormone-induced senescence, and that the rate of lengthening was related positively to the promotion of senescence.

Senescence can also result from DNA damage [20], which can be induced by gamma irradiation [21]. To induce DNA damage, we exposed *Arabidopsis* plants to gamma irradiation at 260 and 1010 Gy and examined the ACLs of all eight lipid classes for 20 days after irradiation. The yellowing of treated leaves showed that their senscence was induced. The growth of the plants was inhibited and they died slowly (Figure S12). The damage under irradiation at 1010 Gy was more severe than that at 260 Gy (Figure S12). The ACLs of all the lipids were highly conserved, apart from that of PS (Figure S13). After 260 Gy irradiation, the ACL of PS continued to increase throughout the 20-day experimental period and had increased by 1.09 C at the end of the period (Figure. 4c and Figure S13). In contrast, after 1010 Gy irradiation, the ACL of PS ceased to increase after 9 days, although it had increased by 1.17 C at this point (Figure. 4c and Figure S13). The maximum rate of lengthening for PS was 0.41±0.07 C/day (Figure. 4d), which was significantly higher than that under normal conditions (Figure. 3b and d). The rate of lengthening after irradiation at 1010 Gy was significantly higher than that after irradiation at 260 Gy, especially during the first 9 days. The earlier cessation of acyl chain lengthening for PS under irradiation at 1010 Gy than under irradiation at 260 Gy could be due to earlier cell death under the former condition (Figure S12). These observations show that the acyl chains of PS lengthened as senescence was induced by DNA damage and that the rate of lengthening was related positively to the severity of damage.

### Changes in ACL of PS under environmental stresses

**Figure 5:**
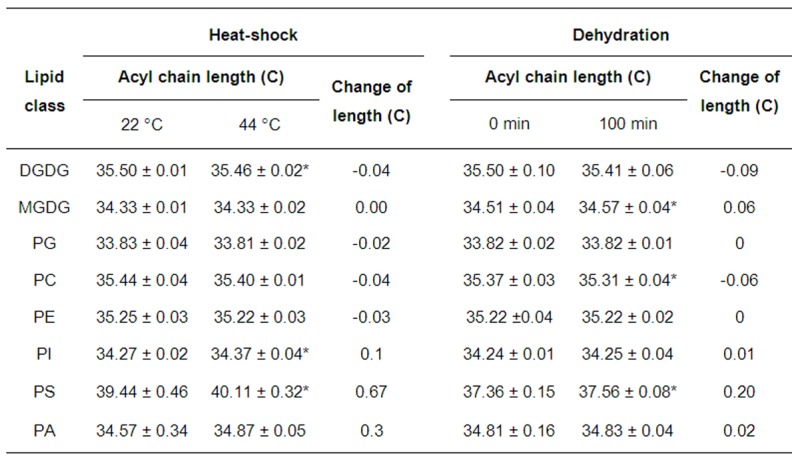
Acyl chains of PS lengthen under stresses in *Arabidopsis*. (Left panel) The acyl chain length of membrane glycerolipids under heat shock at 44°C for 2 h. (**Right panel**) The acyl chain length of membrane glycerolipids under dehydration for 100 min. Values are means ± s.d. (*n* = 5). An asterisk indicates that the value is different from that of the control (*p*<0.05).

Plant growth and senescence are affected by environmental factors, such as temperature [22] and the availability of water [18]. Hence, we investigated whether the acyl chains of PS responded to heat shock (Figure S14) and dehydration. After the plants had been subjected to heat shock for 2 hours and had been dehydrated for 100 minutes, the acyl chains of PS has lengthened significantly by 0.67 and 0.20 C, respectively, whereas the length of the acyl chains of the other lipids varied much less or remained unchanged (Figure. 5). These observations indicated that acyl chains of PS lengthen in response to environmental stresses and can sense corresponding physiological events that have a duration of 1–2 h.

## Discussion

We have shown that ACLs of membrane glycerolipids MGDG, DGDG, PG, PE, PC, PI and PA are conserved among various plant species, during the plant lifespan, and under different environmental stresses and that the ACL of PS increased during the plant lifespan and positively responded to environmental stresses. The increment of ACLs of PS can sense developmental and/or environmental events lasting years, months, days, and hours. Particularly in *Arabidopsis*, total ACL in PS increases from ∼37 to ∼41C during growth and senescence; lengthening stops when plants are close to death, i.e. when ACL of PS reaches 41 C. This is true irrespective of how 41 C is reached, from development or from environmental stress.

Organismal lifespan is determined by the organ lifespan of key functions in animals. In contrast to animals, plants could continuously grow new organs during their lifespan. Even so, however, their lifespans are determined by their organ's lifespan. Yet, the problem is that, the organismal lifespan of different types of plants is determined by the lifespan of different organs. For examples, the lifespan of annual plants is along with their leaf's lifespan; the lifespan of perennial plants is tightly associated with their taproot's lifespan. Given that organ lifespan is correlated with ACL of PS, we therefore suggest that lifespan of a plant species can be measured by the ACL of PS of its particular organ.

PS is one of low abundant membrane lipids (Figure 2 and S3) [23], [24]. PS is located in the inner leaflet of plasma and contributes to maintain membrane asymmetry. Its outward movement disrupts the asymmetry and triggers cell death [25]. Fatty acids of membrane lipids are usually conservative, while fatty acids of storage lipids exhibit diversity [10]. It is not clear why the ACLs of other lipids were conserved and only those of PS increased; we speculate that PS is the only membrane glycerolipid that harbours VLCFAs of greater than 40 C [11], [26]. VLCFAs are thought to be needed for forming highly curved membrane structures [10], thus transgenic plants containing VLCFAs can mimick structural role of sphingolipids, which usually have VLCFAs [27]. Accumulation of VLCFA is deleterious to cells because their special physical and chemical properties can perturb the integrity of membrane structure [10]. It has been proposed that plants have a process to screen-out VLCFA from membranes [28]. In yeast, membrane lipid ergosterol involves in vacuole-vacuole fusion which is required for lifespan extension, however, sphingolipids have no same effects [29]. In animals, PS also plays a unique role in resealing the plasma membrane after it has been damaged [30]. Given that the mechanism by which the plasma membrane is resealed in plants is similar to that by which it is resealed in animals, the lengthening of PS acyl chains could signify an attempt to reseal membranes that have been damaged by senescence and environmental stress. VLCFAs could increase in PS due to their time-dependent accumulation during the lifespan of the plant and due to the response of cells to stress-induced damage. We hypothesise that the tolerance of membrane bilayers to the lengthening of PS acyl chains is limited and that once a critical length is reached, the membrane structure is damaged irreparably and that cause cell death.

In summary, we identified a biomolecule, membrane glycerolipid PS, whose primary structure lengthened during development and natural and artificially– induced senescence of plants. The lengths of the PS acyl chains are limited to ∼41 C and achieving this length appears to be a predictor of the end point of a plant's life. Also the changing length of PS acyl chains between the initial and end stage of development can indicate the lifespan of plants. Thus evidence suggests that ACL of PS could be a molecular scale ruler measuring plant lifespan.

## Supporting Information

Figure S1 **Head-group classes of membrane glycerolipids and the structure of phosphatidylserine (PS).** a,The head group classes of membrane glycerolipids determined, and b The glycerol backbone and acyl chains in the structure of PS.
(TIF)

Figure S2 **The plant species investigated for lipids in the study.** The information of habitats and features of *Saussurea medusa*are taken from website (http://db.kib.ac.cn/eflora/view/search/chs_contents.aspx?name=Saussurea%20medusa%20Maxim; the information on other plant species is from Flora of China.
(TIF)

Figure S3 **The contents (nmol/mg) of lipid molecular species during the development of leaves after germination in** ***Arabidopsis*** **.** Values are means ± s.d. (*n* = 5).
(TIF)

Figure S4 **The ACLs of membrane lipids during the development of leaves in** ***Arabidopsis*** **after germination.** Values are means ±s.d. (*n* = 5). An asterisk indicates that the value is different from that of 24 days after germination (*p*<0.05).
(TIF)

Figure S5 **The composition (mol%) of MGDG molecular species (36∶6 and 34∶6) in eight plant species.** *A. thaliana, S.-L. linearifolia, D. oreades*, and *T. halophila* harbour both 36∶6 and 34∶6MGDG. *S. medusa, V. angularis, M. racemosa*, and *O. sativa* harbour only 36∶6MGDG.
(TIF)

Figure S6 **The range of ACLs of membrane lipids in eight plants.** Values are means ± s.d. (*n* = 5).
(TIF)

Figure S7 **Leaf development of** ***Arabidopsis*** **at 24, 52, 74, and 99 days after germination.**
(TIF)

Figure S8 **The development of** ***Arabidopsis*** **siliques at 15, 20, 25, and 30 days after flowering.**
(TIF)

Figure S9 **The roots of 15-day, 10-month, and 18-month pot-grown and wild perennial (W)** ***Crucihimalaya himalaica*** **.**
(TIF)

Figure S10 **The senescence of detached leaves treated with water, 50 µM ABA, and 50 µM ethephon for 5 days.**
(TIF)

Figure S11 **The acyl chain length of membrane lipids during leaf detachment, ABA, and ethylene-induced senescence in** ***Arabidopsis*** **.** Values are means ±S.D. (*n* = 5). An asterisk indicates that the value is different from that of control (*p*<0.05).
(TIF)

Figure S12 **The senescence of** ***Arabidopsis*** **after 260 and 1010 Gy of gamma-irradiation.** a, Control; b, 260 Gy; and c, 1010Gy.
(TIF)

Figure S13 **The ACLs of membrane lipids during the gamma irradiation-induced senescence in** ***Arabidopsis*** **.** Time is the days after irradiation. Values are means ±s.d. (*n* = 5). An asterisk indicates that the value is different from that of control (*p*<0.05).
(TIF)

Figure S14 **The accumulation of heat-shock protein 70 (HSP70) during head-acclimation and head shock.** Isolated total proteins were conducted by Western.
(TIF)

## References

[1] Finkel T, Holbrook NJ (2000) Oxidants, oxidative stress and the biology of ageing. Nature 408: 239–247. 11089981 10.1038/35041687

[2] Lane MA, Mattison J, Ingram DK, Roth GS (2002) Caloric restriction and aging in primates: Relevance to humans and possible CR mimetics. Microsc Res Tech 59: 335–338. 12424798 10.1002/jemt.10214

[3] Hastie ND, Dempster M, Dunlop MG, Thompson AM, Green DK, et al (1990) Telomere reduction in human colorectal carcinoma and with ageing. Nature 346: 866–868. 2392154 10.1038/346866a0

[4] Allsopp RC, Vaziri H, Patterson C, Goldstein S, Younglai EV, et al (1992) Telomere length predicts replicative capacity of human fibroblasts. Proc Natl Acad Sci U S A 89: 10114–10118. 1438199 10.1073/pnas.89.21.10114 PMC50288

[5] Broun P, Ganal MW, Tanksley SD (1992) Telomeric arrays display high levels of heritable polymorphism among closely related plant varieties. Proc Natl Acad Sci U S A 89: 1354–1357. 1741387 10.1073/pnas.89.4.1354 PMC48448

[6] Kilian A, Stiff C, Kleinhofs A (1995) Barley telomeres shorten during differentiation but grow in callus culture. Proc Natl Acad Sci U S A 92: 9555–9559. 11607583 10.1073/pnas.92.21.9555 PMC40840

[7] Zentgraf U, Hinderhofer K, Kolb D (2000) Specific association of a small protein with the telomeric DNA-protein complex during the onset of leaf senescence in *Arabidopsis thaliana* . Plant Mol Biol 42: 429–438. 10798613 10.1023/a:1006324008600

[8] Marba N, Duarte CM, Agusti S (2007) Allometric scaling of plant life history. Proc Natl Acad Sci U S A 104: 15777–15780. 17890320 10.1073/pnas.0703476104 PMC1989662

[9] Kim J, Jung JH, Lee SB, Go YS, Kim HJ, et al (2013) Arabidopsis 3-ketoacyl-coenzyme A synthase9 is involved in the synthesis of tetracosanoic acids as precursors of cuticular waxes, suberins, sphingolipids, and phospholipids. Plant Physiology 162: 567–580. 23585652 10.1104/pp.112.210450 PMC3668053

[10] Millar AA, Smith MA, Kunst L (2000) All fatty acids are not equal: discrimination in plant membrane lipids. Trends Plant Sci 5: 95–101. 10707074 10.1016/s1360-1385(00)01566-1

[11] Devaiah SP, Roth MR, Baughman E, Li M, Tamura P, et al (2006) Quantitative profiling of polar glycerolipid species from organs of wild-type Arabidopsis and a phospholipase Dalpha1 knockout mutant. Phytochemistry 67: 1907–1924. 16843506 10.1016/j.phytochem.2006.06.005

[12] Buchanan B, Gruissem W, Jones R (2002) Biochemistry & Molecular Biology of Plants, pp. 499. John Wiley & Sons.

[13] Welti R, Li W, Li M, Sang Y, Biesiada H, et al (2002) Profiling membrane lipids in plant stress responses. Role of phospholipase D alpha in freezing-induced lipid changes in Arabidopsis. J Biol Chem 277: 31994–32002. 12077151 10.1074/jbc.M205375200

[14] Zheng G, Tian B, Zhang F, Tao F, Li W (2011) Plant adaptation to frequent alterations between high and low temperatures: remodelling of membrane lipids and maintenance of unsaturation levels. Plant Cell Environ 34: 1431–1442. 21486310 10.1111/j.1365-3040.2011.02341.x PMC3980542

[15] Tocquin P, Corbesier L, Havelange A, Pieltain A, Kurtem E, et al (2003) A novel high efficiency, low maintenance, hydroponic system for synchronous growth and flowering of *Arabidopsis thaliana* . BMC Plant Biol 3: 2. 12556248 10.1186/1471-2229-3-2 PMC150571

[16] Fan L, Zheng S, Wang X (1997) Antisense suppression of phospholipase D alpha retards abscisic acid- and ethylene-promoted senescence of postharvest Arabidopsis leaves. Plant Cell 9: 2183–2196. 9437863 10.1105/tpc.9.12.2183 PMC157067

[17] Choe HT, Whang M (1986) Effects of ethephon on aging and photosynthetic activity in isolated chloroplasts. Plant Physiol 80: 305–309. 16664618 10.1104/pp.80.2.305 PMC1075109

[18] Katagiri T, Takahashi S, Shinozaki K (2001) Involvement of a novel Arabidopsis phospholipase D, AtPLDdelta, in dehydration-inducible accumulation of phosphatidic acid in stress signalling. Plant J 26: 595–605. 11489173 10.1046/j.1365-313x.2001.01060.x

[19] Welti R, Wang X (2004) Lipid species profiling: a high-throughput approach to identify lipid compositional changes and determine the function of genes involved in lipid metabolism and signaling. Curr Opin Plant Biol 7: 337–344. 15134756 10.1016/j.pbi.2004.03.011

[20] Collado M, Blasco MA, Serrano M (2007) Cellular senescence in cancer and aging. Cell 130: 223–233. 17662938 10.1016/j.cell.2007.07.003

[21] Boubriak, II, Grodzinsky DM, Polischuk VP, Naumenko VD, Gushcha NP, et al (2008) Adaptation and impairment of DNA repair function in pollen of *Betula verrucosa* and seeds of *Oenothera biennis* from differently radionuclide-contaminated sites of Chernobyl. Ann Bot 101: 267–276. 17981881 10.1093/aob/mcm276 PMC2711018

[22] Hong SW, Vierling E (2000) Mutants of *Arabidopsis thaliana* defective in the acquisition of tolerance to high temperature stress. Proc Natl Acad Sci U S A 97: 4392–4397. 10760305 10.1073/pnas.97.8.4392 PMC18252

[23] Li W, Wang R, Li M, Li L, Wang C, et al (2008) Differential degradation of extraplastidic and plastidic lipids during freezing and post-freezing recovery in *Arabidopsis thaliana* . J Biol Chem 283: 461–468. 17962199 10.1074/jbc.M706692200

[24] Zhang X, Wang R, Zhang F, Tan F, LI W (2013) Lipid profiling and tolerance to low-temperature stress in *Thellungiella salsuginea* in comparison with *Arabidopsis thaliana* . Biologia Plantarum 57: 149–153.

[25] Fadok VA, Voelker DR, Campbell PA, Cohen JJ, Bratton DL, et al (1992) Exposure of phosphatidylserine on the surface of apoptotic lymphocytes triggers specific recognition and removal by macrophages. J Immunol 148: 2207–2216. 1545126

[26] Yamaoka Y, Yu Y, Mizoi J, Fujiki Y, Saito K, et al (2011) PHOSPHATIDYLSERINE SYNTHASE1 is required for microspore development in *Arabidopsis thaliana* . Plant J 67: 648–661. 21554450 10.1111/j.1365-313X.2011.04624.x

[27] Schneiter R, Kohlwein SD (1997) Organelle structure, function, and inheritance in yeast: a role for fatty acid synthesis? Cell 88: 431–434. 9038333 10.1016/s0092-8674(00)81882-6

[28] Murphy DJ (2005) Plant lipids: biology, utilisation, and manipulation. Blackwell Pub: CRC Press, Oxford Boca Raton, FL. pp81.

[29] Tang F, Watkins JW, Bermudez M, Gray R, Gaban A, et al (2008) A life-span extending form of autophagy employs the vacuole-vacuole fusion machinery. Autophagy 4: 874–886. 18690010 10.4161/auto.6556

[30] Bouter A, Gounou C, Berat R, Tan S, Gallois B, et al (2011) Annexin-A5 assembled into two-dimensional arrays promotes cell membrane repair. Nat Commun 2: 270. 21468022 10.1038/ncomms1270 PMC3104517

