# Supplementary figures and images for: Acyl Chain Length of Phosphatidylserine Is Correlated with Plant Lifespan
Source: PLoS One. 2014 Jul 24;9(7):e103227. doi: 10.1371/journal.pone.0103227 (PMC4110022; doi:10.1371/journal.pone.0103227)

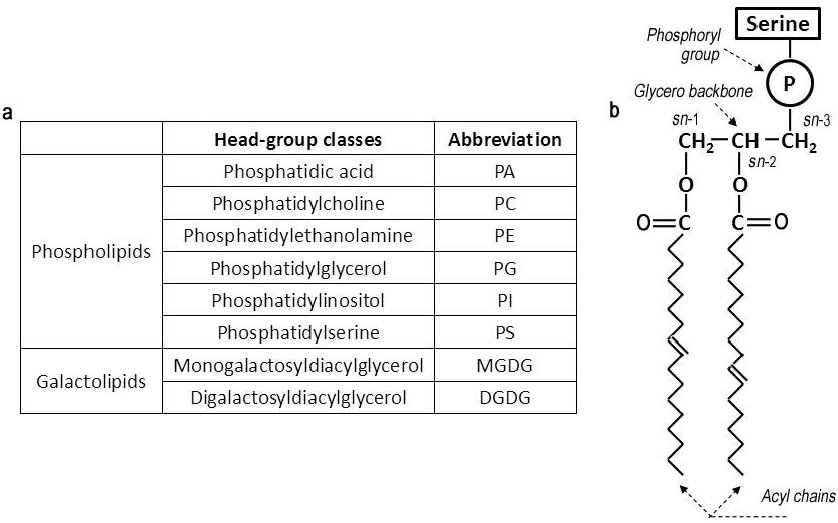

Supplement: Figure S1 — Head-group classes of membrane glycerolipids and the structure of phosphatidylserine (PS). a,The head group classes of membrane glycerolipids determined, and b The glycerol backbone and acyl chains in the structure of PS. (TIF) [file pone.0103227.s001.tif]

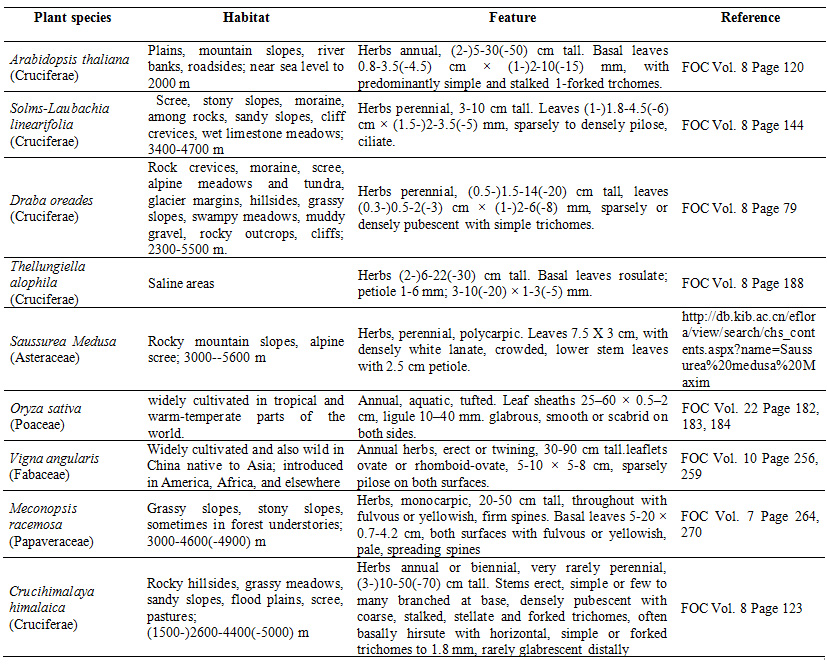

Supplement: Figure S2 — The plant species investigated for lipids in the study. The information of habitats and features of Saussurea medusaare taken from website (http://db.kib.ac.cn/eflora/view/search/chs_contents.aspx?name=Saussurea%20medusa%20Maxim; the information on other plant species is from Flora of China. (TIF) [file pone.0103227.s002.tif]

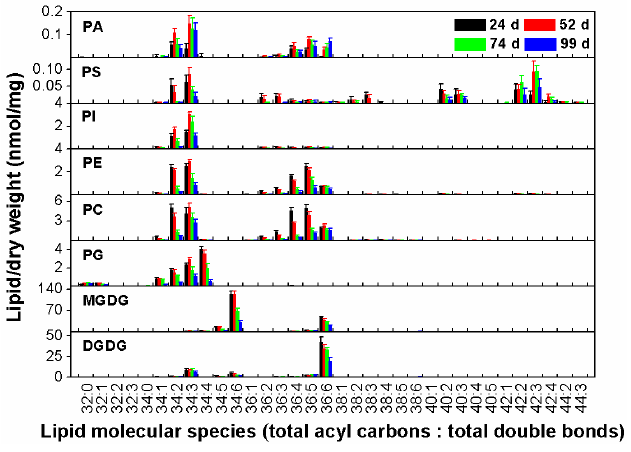

Supplement: Figure S3 — The contents (nmol/mg) of lipid molecular species during the development of leaves after germination in Arabidopsis . Values are means ± s.d. (n = 5). (TIF) [file pone.0103227.s003.tif]

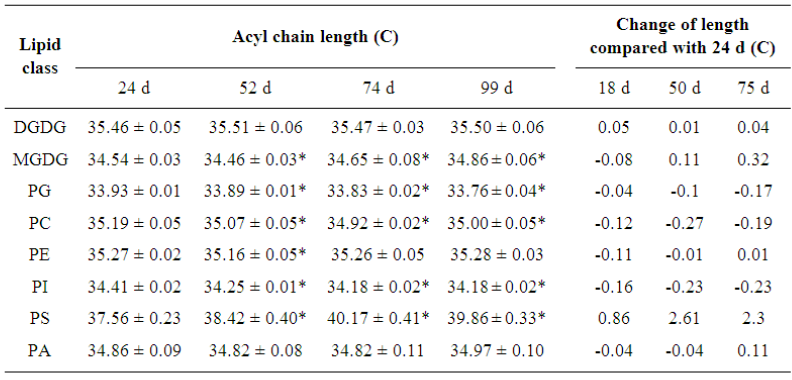

Supplement: Figure S4 — The ACLs of membrane lipids during the development of leaves in Arabidopsis after germination. Values are means ±s.d. (n = 5). An asterisk indicates that the value is different from that of 24 days after germination (p<0.05). (TIF) [file pone.0103227.s004.tif]

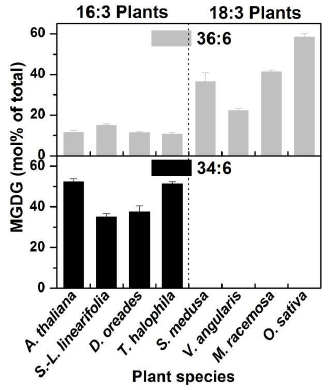

Supplement: Figure S5 — The composition (mol%) of MGDG molecular species (36∶6 and 34∶6) in eight plant species. A. thaliana, S.-L. linearifolia, D. oreades, and T. halophila harbour both 36∶6 and 34∶6MGDG. S. medusa, V. angularis, M. racemosa, and O. sativa harbour only 36∶6MGDG. (TIF) [file pone.0103227.s005.tif]

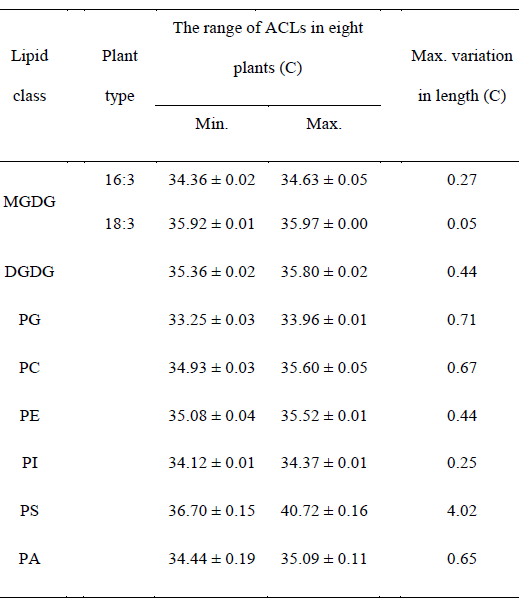

Supplement: Figure S6 — The range of ACLs of membrane lipids in eight plants. Values are means ± s.d. (n = 5). (TIF) [file pone.0103227.s006.tif]

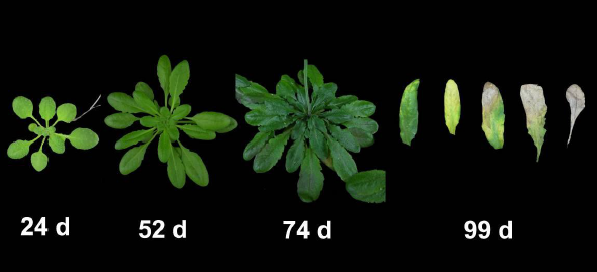

Supplement: Figure S7 — Leaf development of Arabidopsis at 24, 52, 74, and 99 days after germination. (TIF) [file pone.0103227.s007.tif]

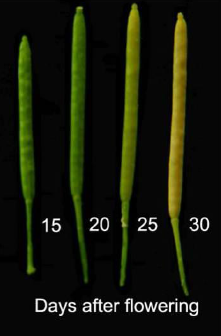

Supplement: Figure S8 — The development of Arabidopsis siliques at 15, 20, 25, and 30 days after flowering. (TIF) [file pone.0103227.s008.tif]

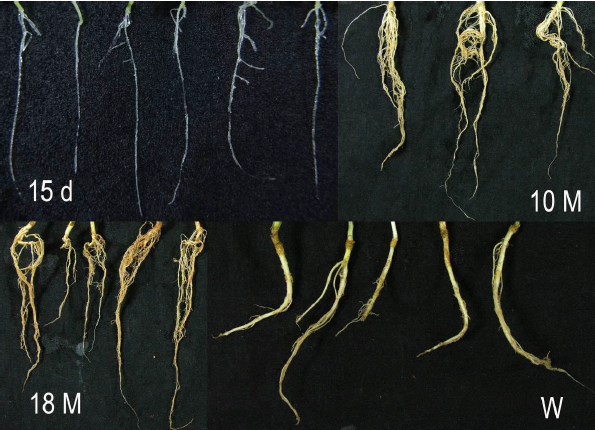

Supplement: Figure S9 — The roots of 15-day, 10-month, and 18-month pot-grown and wild perennial (W) Crucihimalaya himalaica . (TIF) [file pone.0103227.s009.tif]

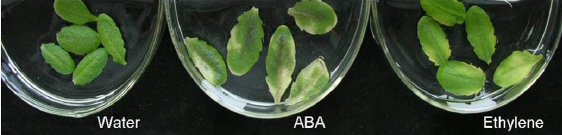

Supplement: Figure S10 — The senescence of detached leaves treated with water, 50 µM ABA, and 50 µM ethephon for 5 days. (TIF) [file pone.0103227.s010.tif]

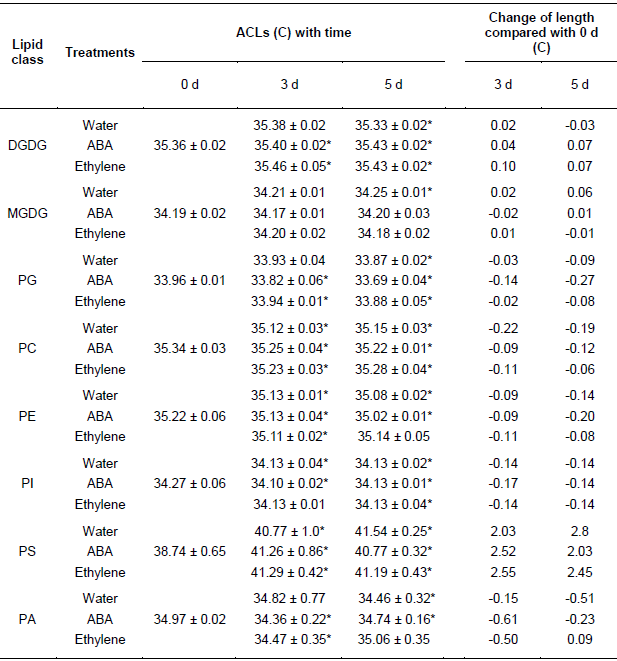

Supplement: Figure S11 — The acyl chain length of membrane lipids during leaf detachment, ABA, and ethylene-induced senescence in Arabidopsis . Values are means ±S.D. (n = 5). An asterisk indicates that the value is different from that of control (p<0.05). (TIF) [file pone.0103227.s011.tif]

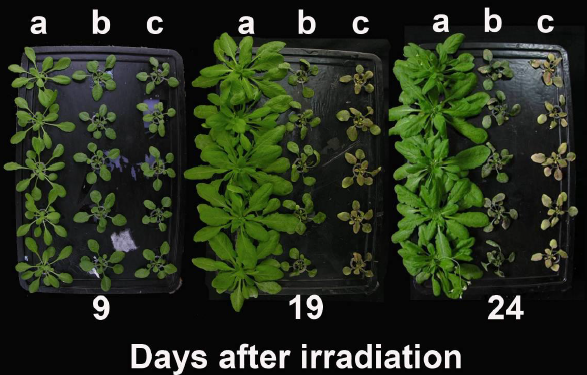

Supplement: Figure S12 — The senescence of Arabidopsis after 260 and 1010 Gy of gamma-irradiation. a, Control; b, 260 Gy; and c, 1010Gy. (TIF) [file pone.0103227.s012.tif]

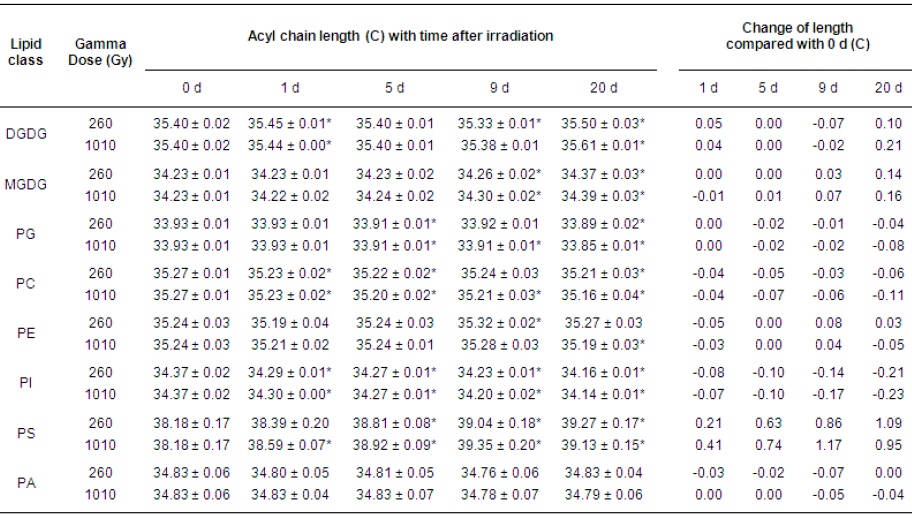

Supplement: Figure S13 — The ACLs of membrane lipids during the gamma irradiation-induced senescence in Arabidopsis . Time is the days after irradiation. Values are means ±s.d. (n = 5). An asterisk indicates that the value is different from that of control (p<0.05). (TIF) [file pone.0103227.s013.tif]

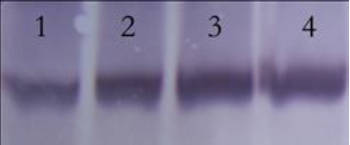

Supplement: Figure S14 — The accumulation of heat-shock protein 70 (HSP70) during head-acclimation and head shock. Isolated total proteins were conducted by Western. (TIF) [file pone.0103227.s014.tif]
